# Revolutionizing Oral Rehabilitation With Modified Andrew’s Bridge for Alveolar Bone Defect: A Case Report

**DOI:** 10.7759/cureus.67075

**Published:** 2024-08-17

**Authors:** Sidhartha Tomar, Ayush Kumar

**Affiliations:** 1 Prosthodontics and Crown and Bridge, Subharti Dental College and Hospital, Swami Vivekanand Subharti University, Meerut, IND

**Keywords:** removable and fixed prosthesis, cad- cam, precision attachment, alveolar bone loss, partial denture

## Abstract

Within the realm of prosthodontics, the challenge of replacing multiple missing teeth is a complex one, compounded by patient’s preference for fixed prostheses over removable ones owing to their superior aesthetic and functional attributes. However, the feasibility of fixed prostheses diminishes in scenarios marked by compromised remaining dentition and defects in edentulous regions. To navigate these challenges effectively, the Andrew’s bridge emerges as a compelling solution, integrating both fixed and removable components. This approach, particularly adept at addressing extensive alveolar bone defects, offers a synthesis of advantages including enhanced phonetics, hygiene, aesthetics and function. This article details a case report that outlines the digital fabrication process of an Andrews Bridge used to treat a maxillary anterior ridge defect.

## Introduction

Alveolar bone is the bony portion of the mandible or maxilla where the roots of the teeth are held by the fibers of the periodontal ligament [1]. Defects in the alveolar bone can result from various causes, including congenital conditions such as clefts, and acquired factors like trauma or tumor resection. These defects can compromise aesthetics and function, affect the fit and stability of dental prostheses and impact overall oral health. In dentistry, addressing significant alveolar bone defects presents considerable challenges [2]. However, advancements in treatment modalities offer tailored solutions for such cases. Utilizing Siebert's classification from 1983, which categorizes bone defects and CAD/CAM technology introduced by Professor François Duret in 1971, precise and customized prostheses, such as Andrew’s bridges, can be fabricated to restore both function and aesthetics effectively [3]. The fixed removable Andrew's system was first introduced by Dr. James Andrews of Amite, Louisiana, in 1975 [4]. It is a combination of a fixed partial denture incorporating a connecting bar with a removable partial denture that replaces teeth within the bar area. The Andrew’s Bridge, while not as commonly used as conventional fixed prostheses or implant-supported options, is still a viable solution in certain clinical scenarios, particularly in cases of significant alveolar bone loss where traditional fixed prostheses are not feasible. Its usage varies by region, with some practitioners favouring it for its ability to address specific anatomic challenges. However, its adoption is generally more limited compared to other prosthodontic solutions, largely due to the specialized nature of the cases it addresses and the availability of alternative treatment options such as dental implants.

## Case presentation

This case report describes the fabrication of a modified Andrew’s bridge to treat a male patient with Siebert’s Class III anterior ridge defect, which occurred due to midfacial trauma in a road traffic accident. It explores the utilization of advanced technology to address a complex dental challenge. The patient was counseled regarding orthodontic treatment beforehand, but his primary concern was the rehabilitation of the upper anterior region for aesthetic reasons. Respecting his concern, we planned our treatment accordingly.

**Figure 1:**
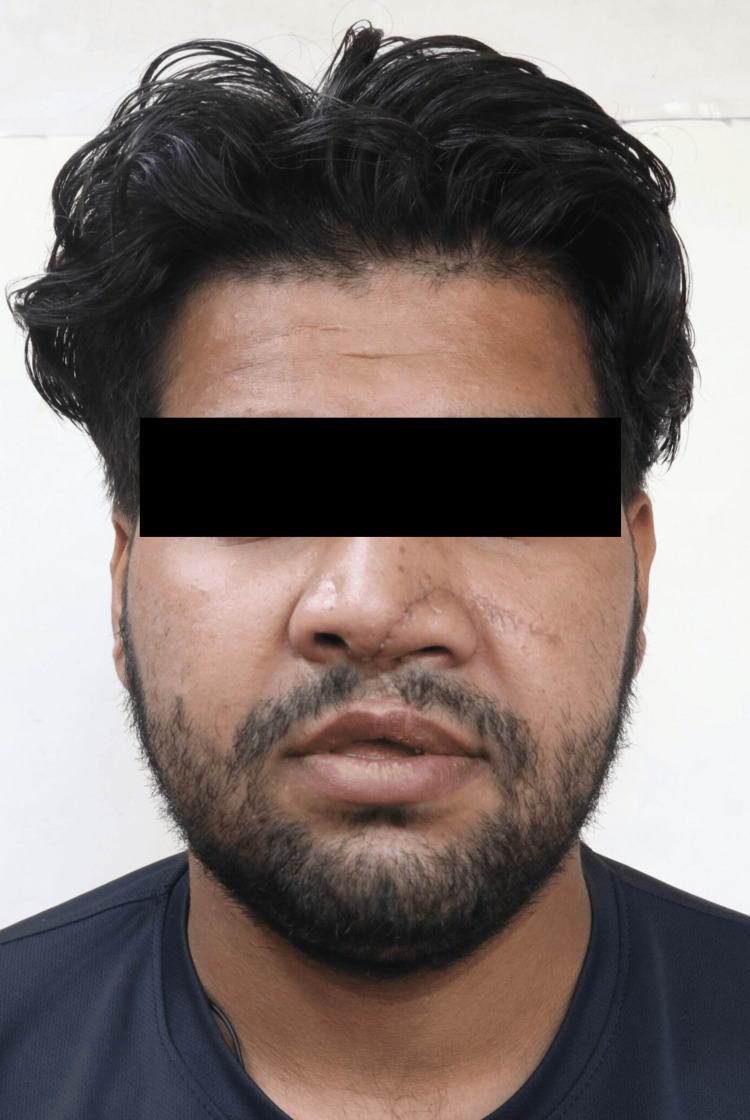
Frontal view Extraoral frontal view of the face showing a midline shift towards the right side.

**Figure 2:**
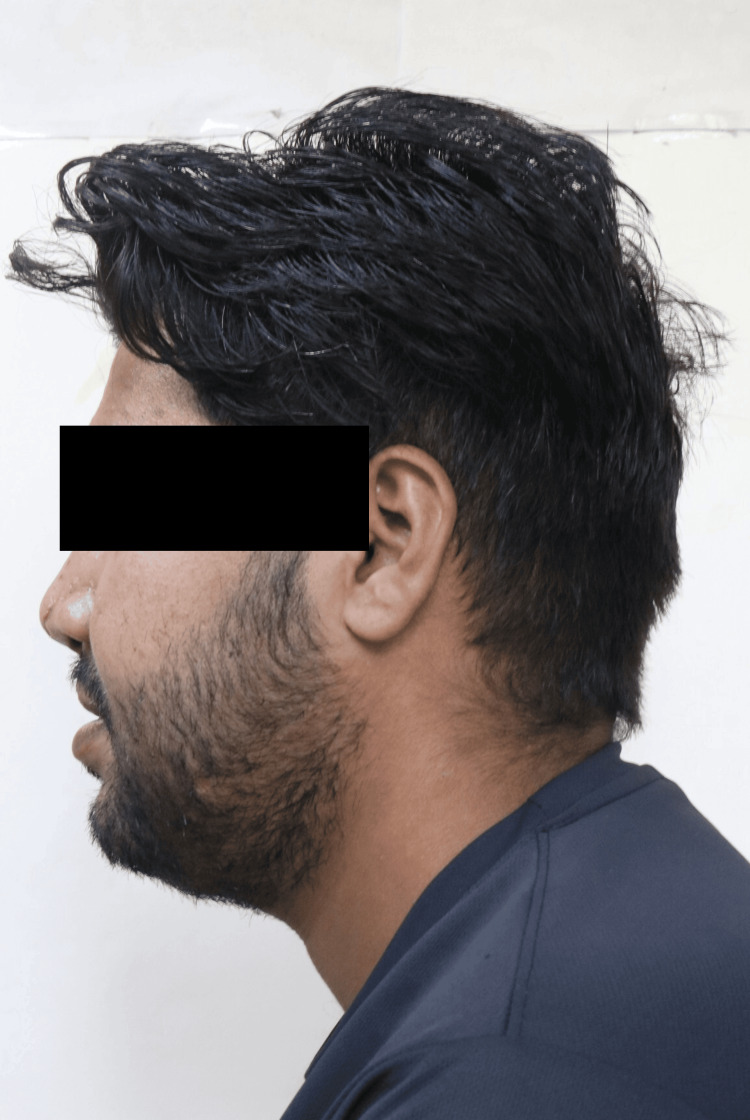
Left lateral profile Left lateral profile is straight.

**Figure 3:**
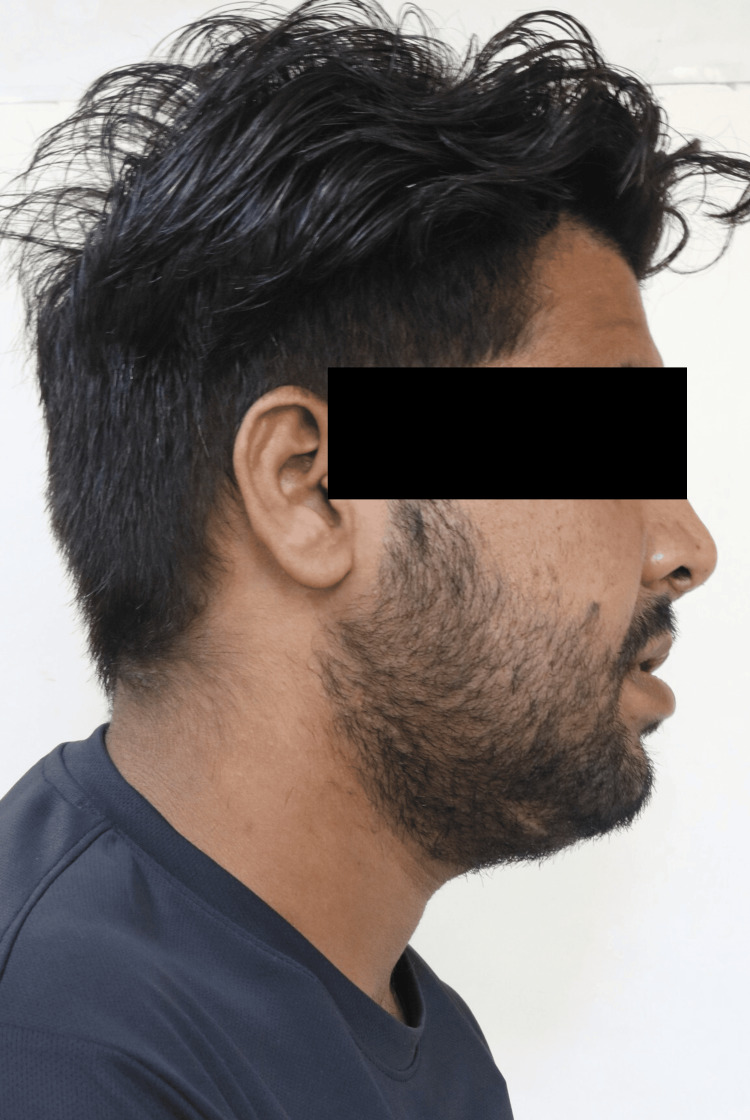
Right lateral profile Right lateral profile is straight.

**Figure 4:**
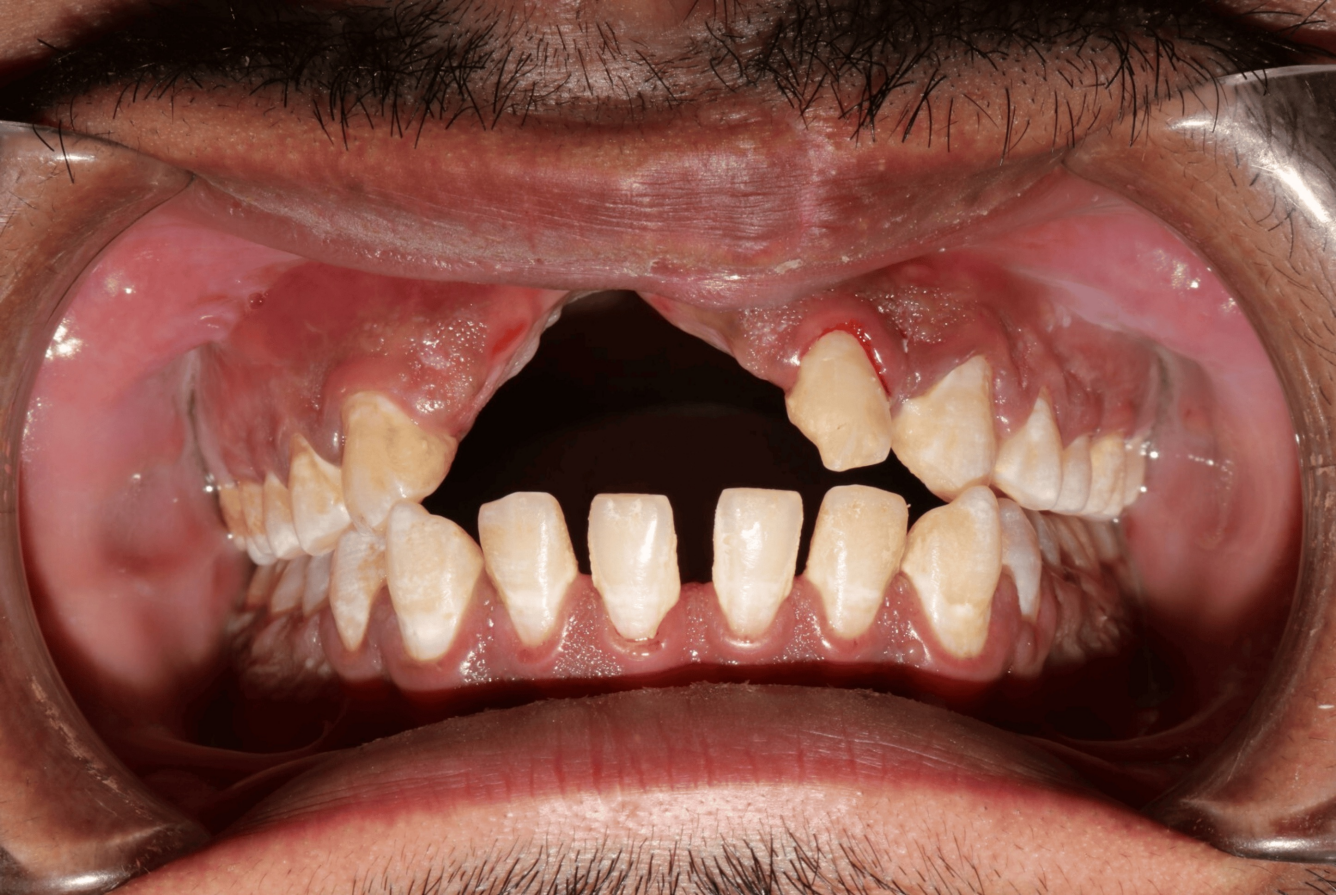
Intraoral view Intraoral view showing missing teeth 12, 11, and 21, along with an alveolar bone defect in the maxillary anterior region having a vertical component measuring approximately 14mm.

**Figure 5:**
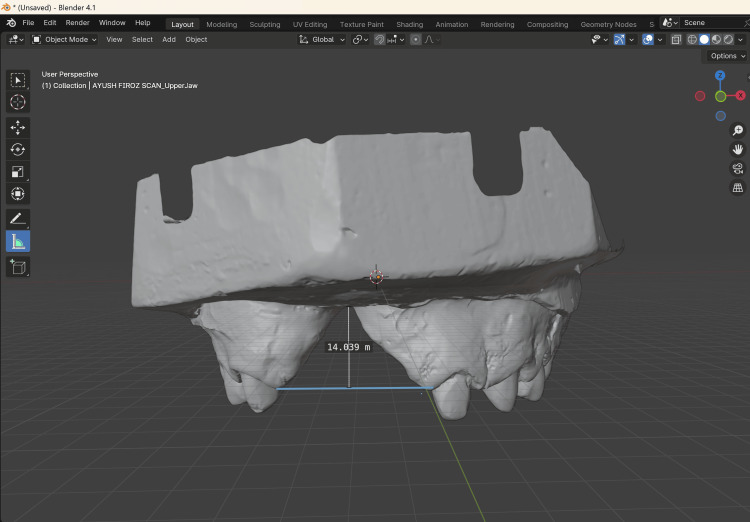
Vertical component of the defect Vertical component of the defect was 14mm measured using Blender 4.1 software.

**Figure 6:**
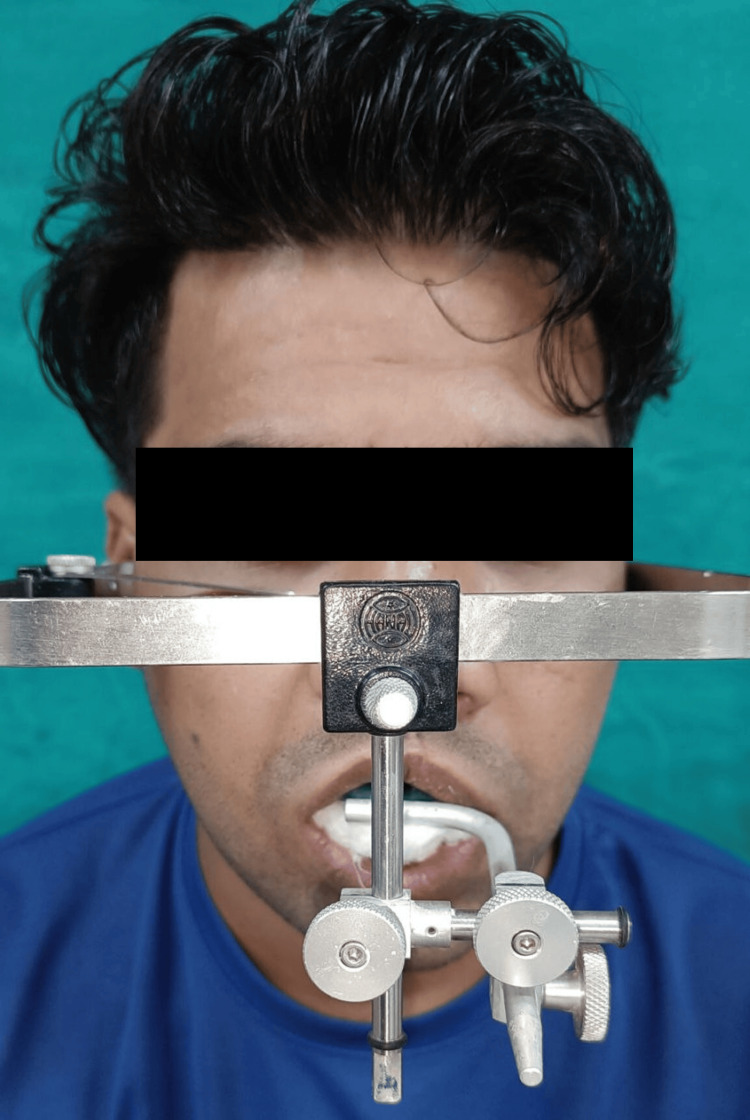
Orientation jaw relation Orientation jaw relation was recorded with a Hanau Springbow facebow.

**Figure 7:**
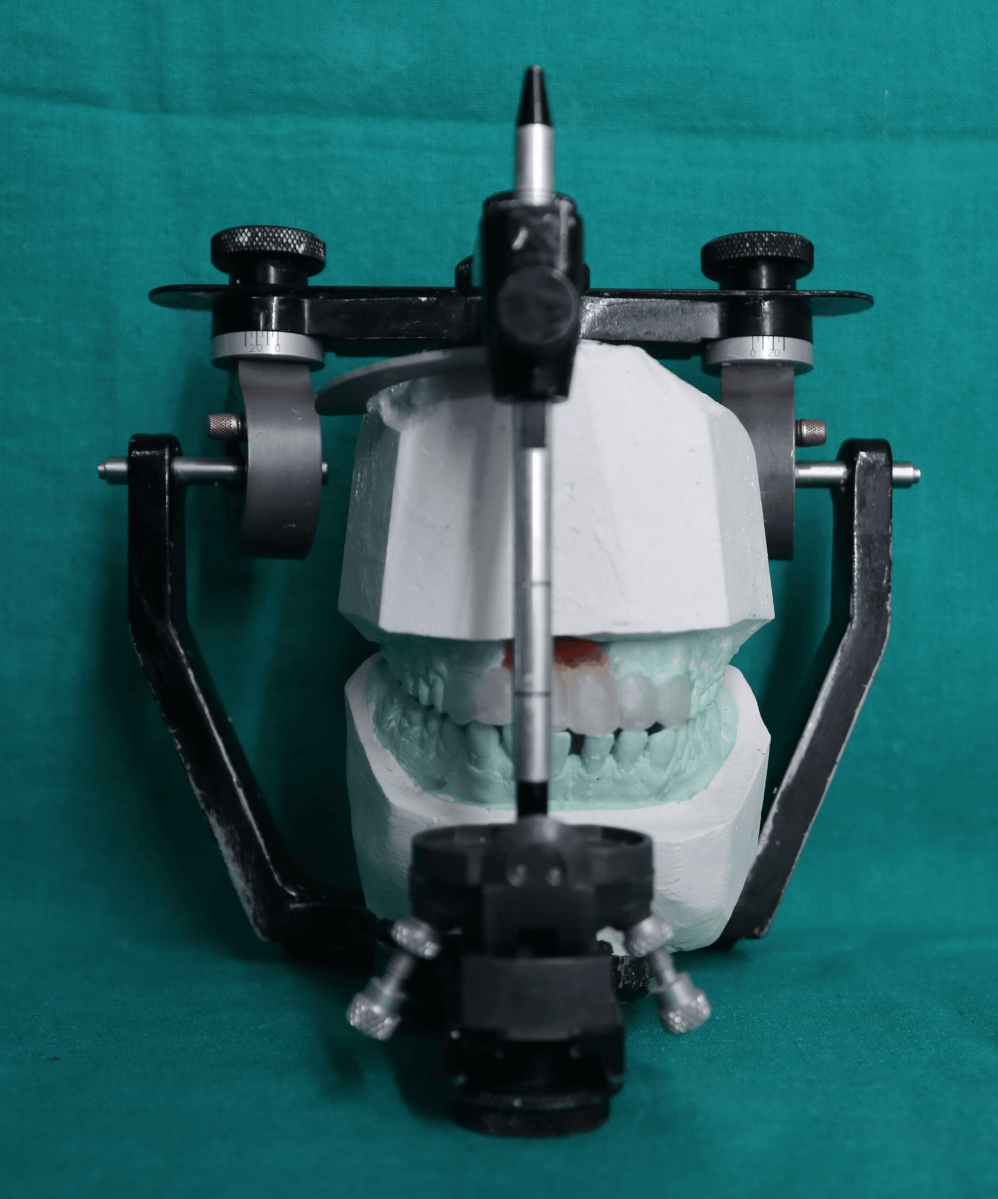
Diagnostic mockup Diagnostic mockup performed prior to mouth preparations using mockup wax.

**Figure 8:**
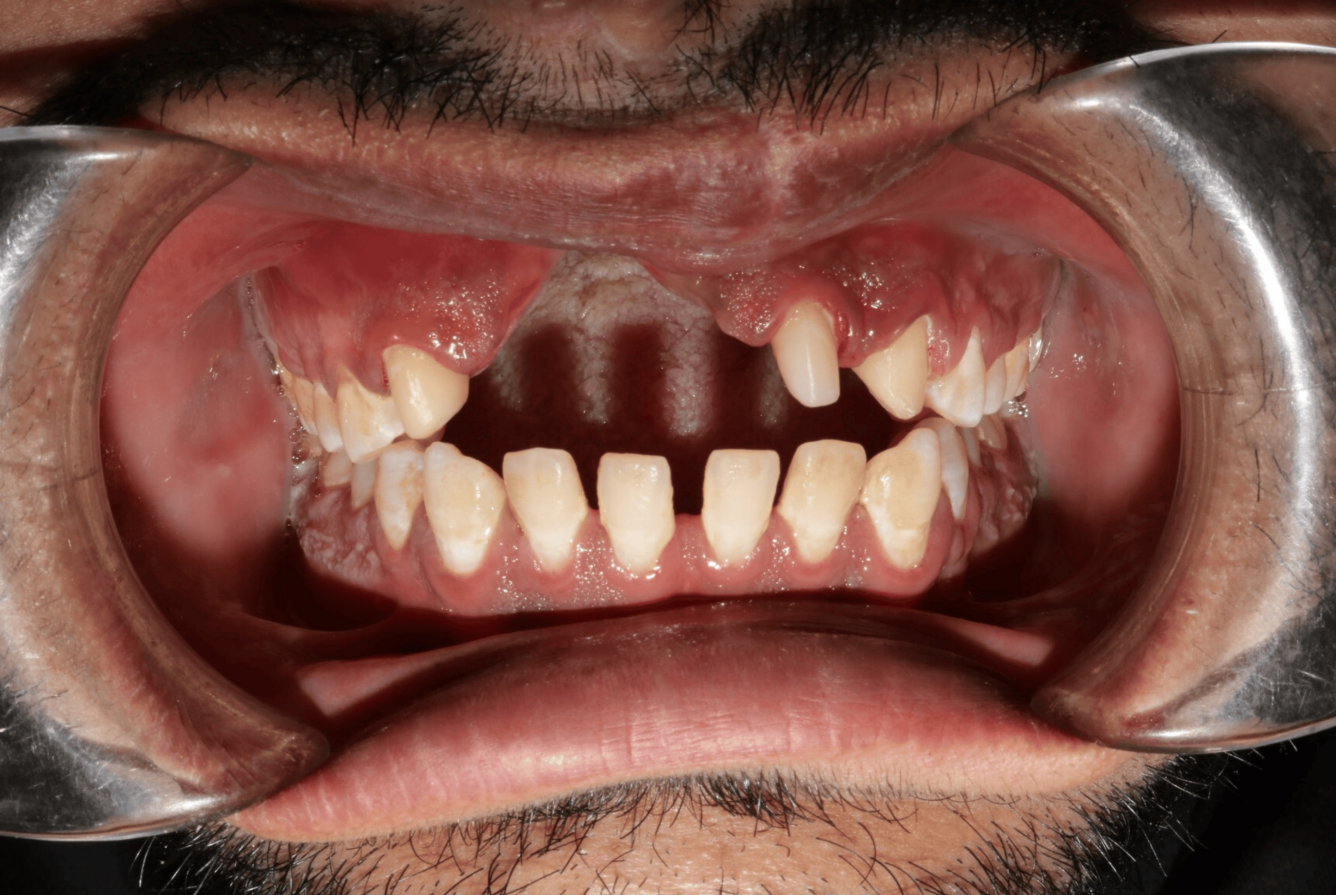
Mouth preparation Teeth preparation: 13, 22, and 23 for porcelain fused to metal crowns constituting the fixed portion of Andrew’s system.

During the extraoral examination, a midline shift towards the right side was observed following the surgical procedure (Figure 1), while both the left (Figure 2) and right (Figure 3) lateral profiles remained straight. The intraoral examination revealed missing teeth 12, 11, and 21, along with an alveolar bone defect in the maxillary anterior region (Figure 4) and a vertical component measuring approximately 14mm using Blender 4.1 software (Figure 5). Diagnostic impressions for the maxillary and mandibular arches were made using irreversible hydrocolloid. The orientation jaw relation was recorded with a Hanau Springbow facebow (Figure 6) and transferred to a Hanau Wide-Vue semi-adjustable articulator. A diagnostic mockup was performed prior to mouth preparations for patient education and treatment planning (Figure 7). Teeth 13, 22, and 23 were prepared for porcelain fused to metal crowns, constituting the fixed portion of Andrew’s system (Figure 8). Following gingival retraction, a definitive impression was made using addition silicone elastomer.

**Figure 9:**
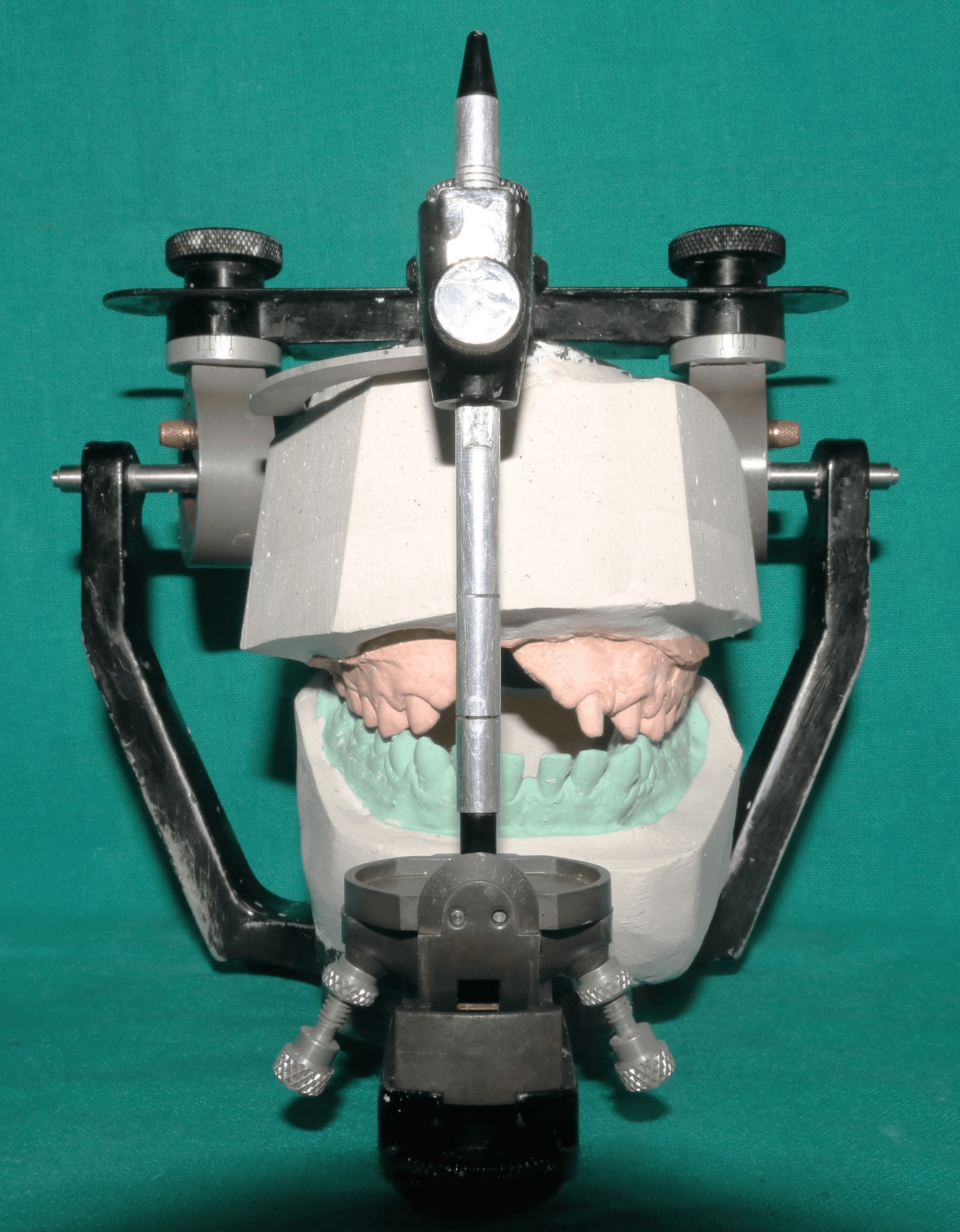
Mounting of definitive casts on semi-adjustable articulator Definitive casts with prepared teeth mounted on semi-adjustable articulator.

**Figure 10:**
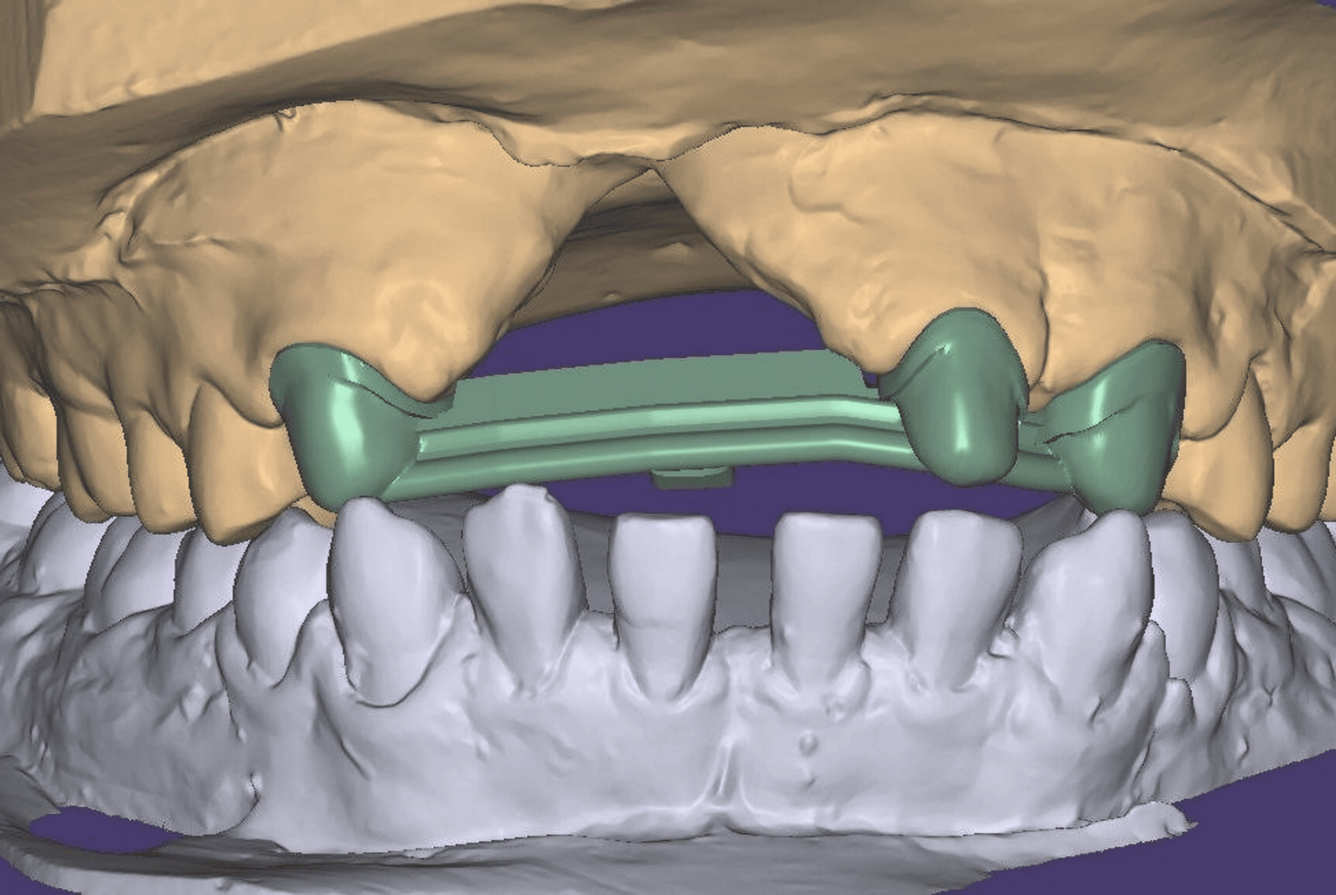
Designed fixed portion of Andrew's system using Exocad software - Frontal view Frontal view of CAD-designed fixed prosthetic part designed using Exocad software.

**Figure 11:**
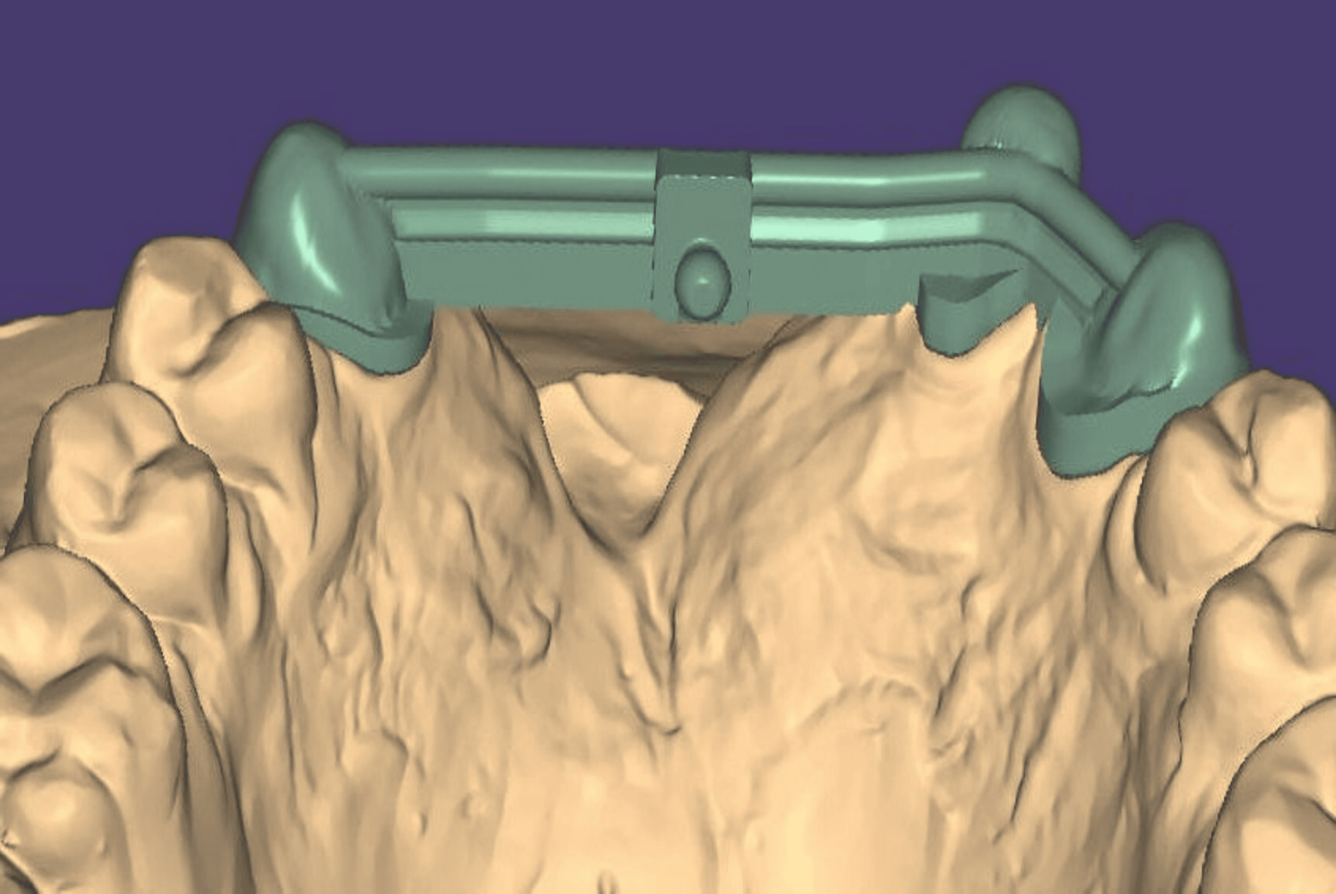
Designed fixed portion of Andrew's system using Exocad software - Palatal view Palatal view of CAD-designed fixed prosthetic part designed using Exocad software.

**Figure 12:**
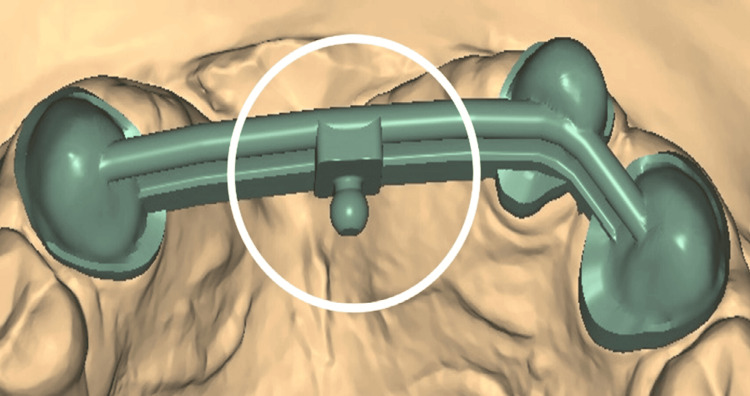
Modification of Andrew's bridge system Incorporation of an attachment on the palatal aspect of the Hader bar for stabilization of the prosthesis.

**Figure 13:**
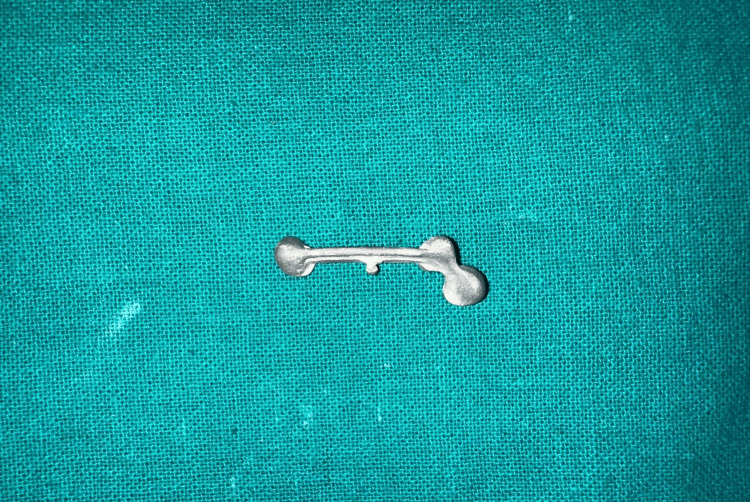
Milled Co-Cr alloy fixed prosthesis with palatal modified attachment Prosthesis fabricated by computer-assisted milling (CAM) of a Co-Cr alloy disc.

**Figure 14:**
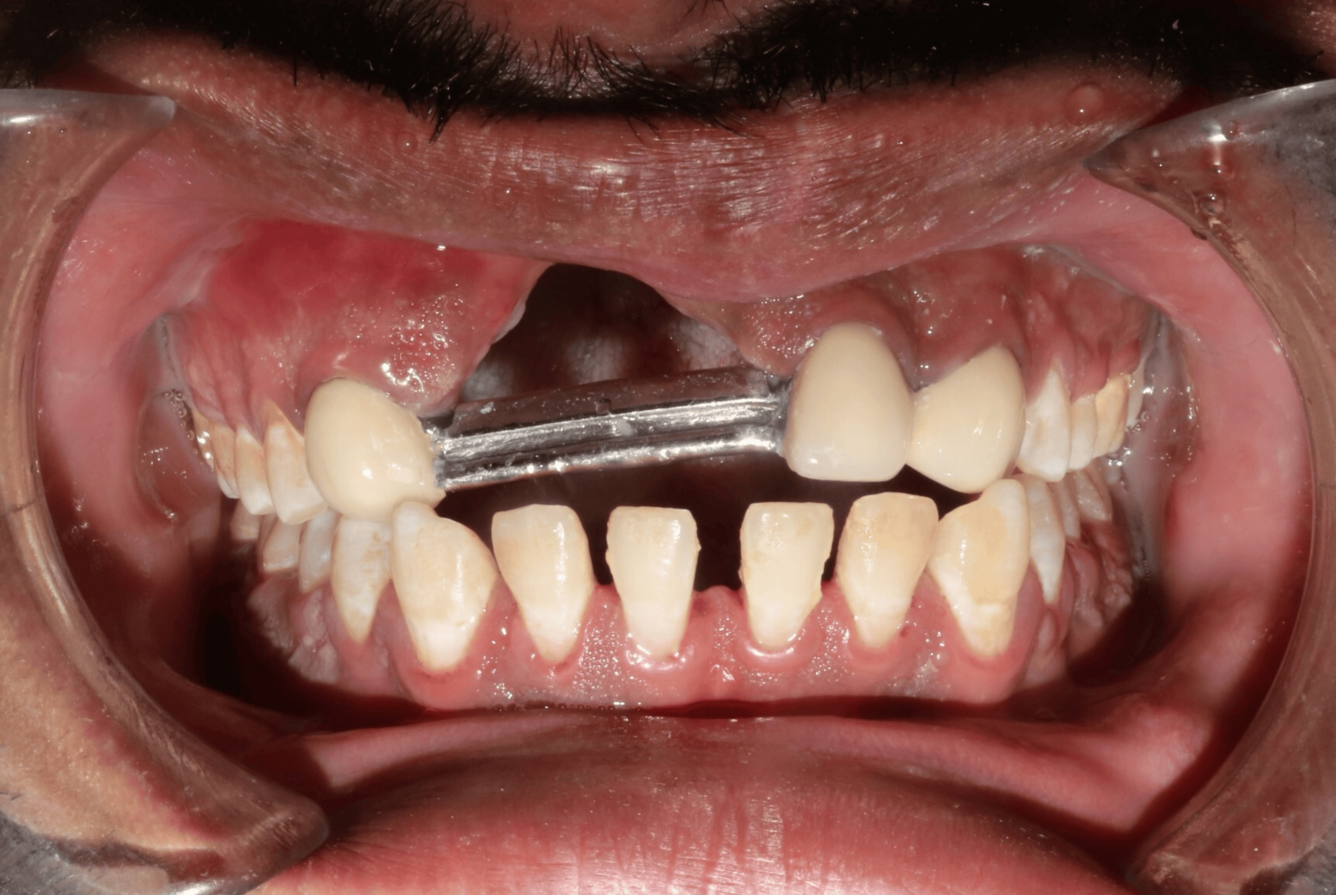
Try-in of fixed prosthesis Try-in of ceramic-layered fixed prosthesis.

**Figure 15:**
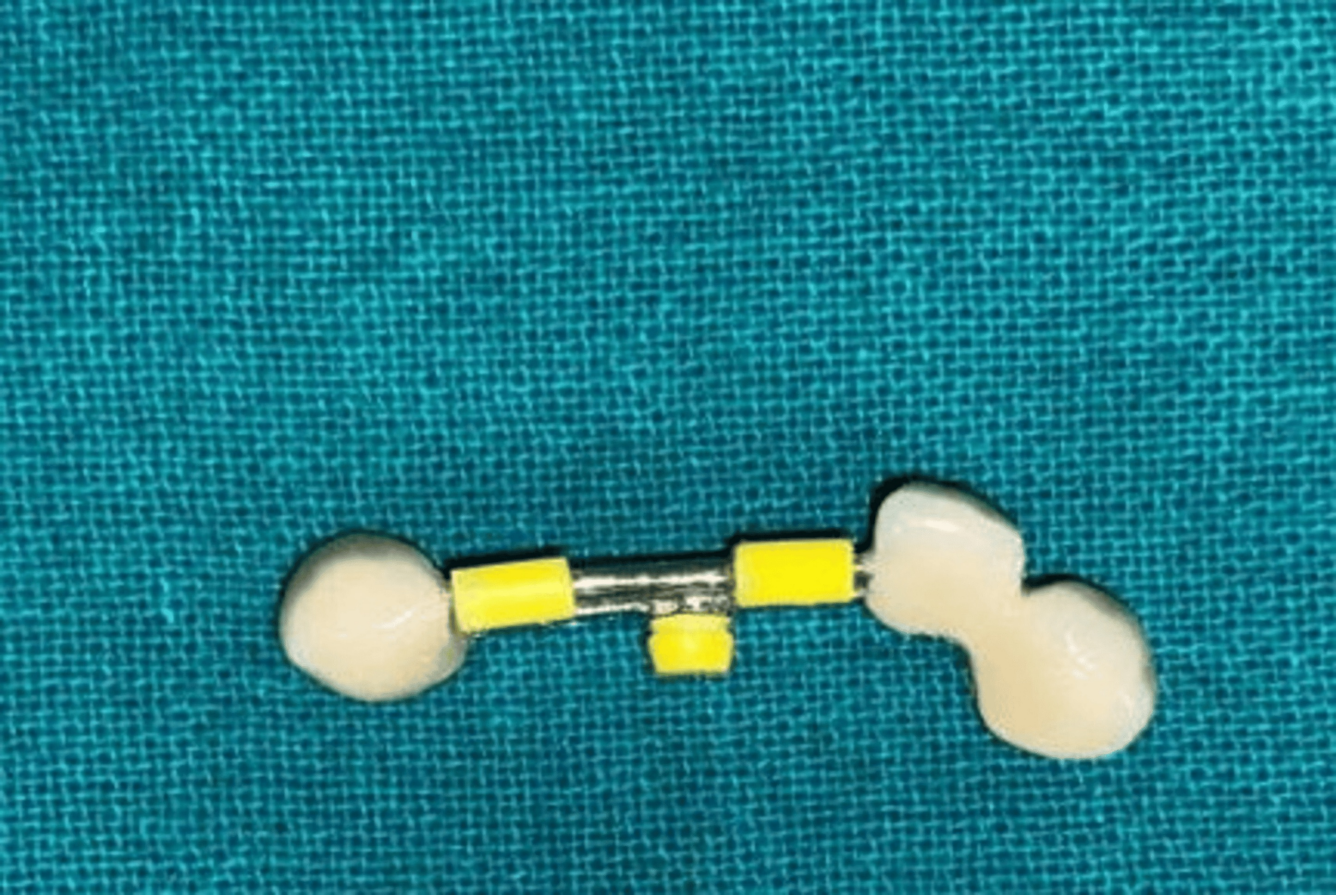
Fixed prosthesis with matrix part attached for extraoral scanning Extraoral scanning of fixed prosthesis along with preci-horix and preci-sagix female components attached for designing the removable cast partial denture.

The facebow was recorded again, and the record was transferred for mounting the definitive cast on a semi-adjustable articulator (Figure 9). The cast models were digitally scanned using an extraoral scanner, followed by computer-aided design (CAD) of the fixed part of the prosthesis, including metal copings and a Hader bar with a modification (Figures 10, 11). The modification in the Andrew’s system involved incorporating an attachment (2.2mm ball attachment) on the palatal aspect of the Hader bar for stabilization of the prosthesis (Figure 12), whereas the female component consisted of a yellow cap incorporated in the removable cast partial denture. The prosthesis was fabricated by computer-assisted milling (CAM) of a Co-Cr alloy disc (Figure 13). The metal framework was tried to check for the marginal fit of the copings, and once verified, porcelain layering was performed (Figure 14). After fabricating the fixed portion, the cast model was scanned extraorally again, along with the prosthesis, incorporating preci-horix and preci-sagix female components for designing the removable cast partial denture (Figure 15).

**Figure 16:**
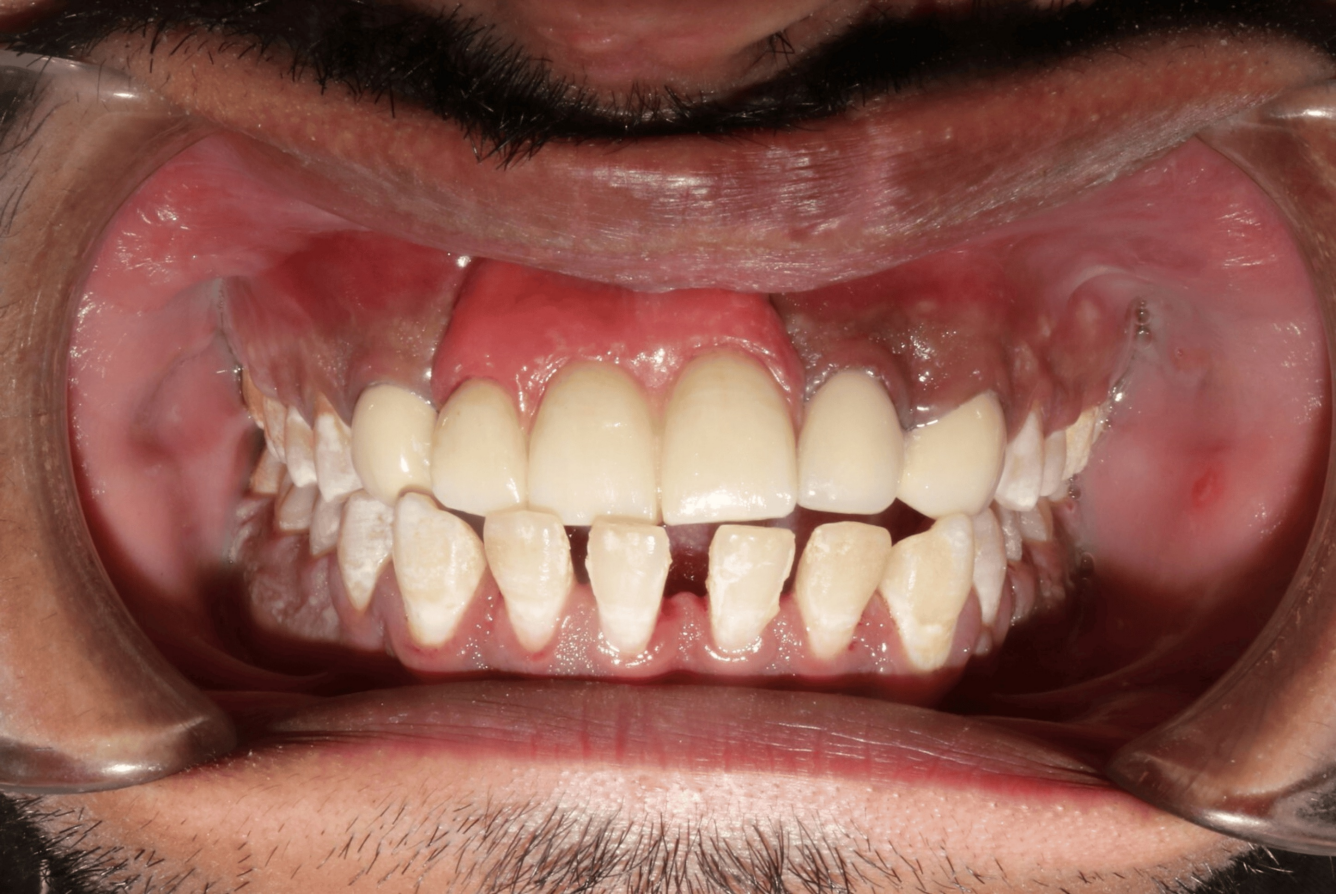
Insertion of Andrew's bridge system Cementation of fixed prosthesis, followed by the insertion of the cast partial denture.

**Figure 17:**
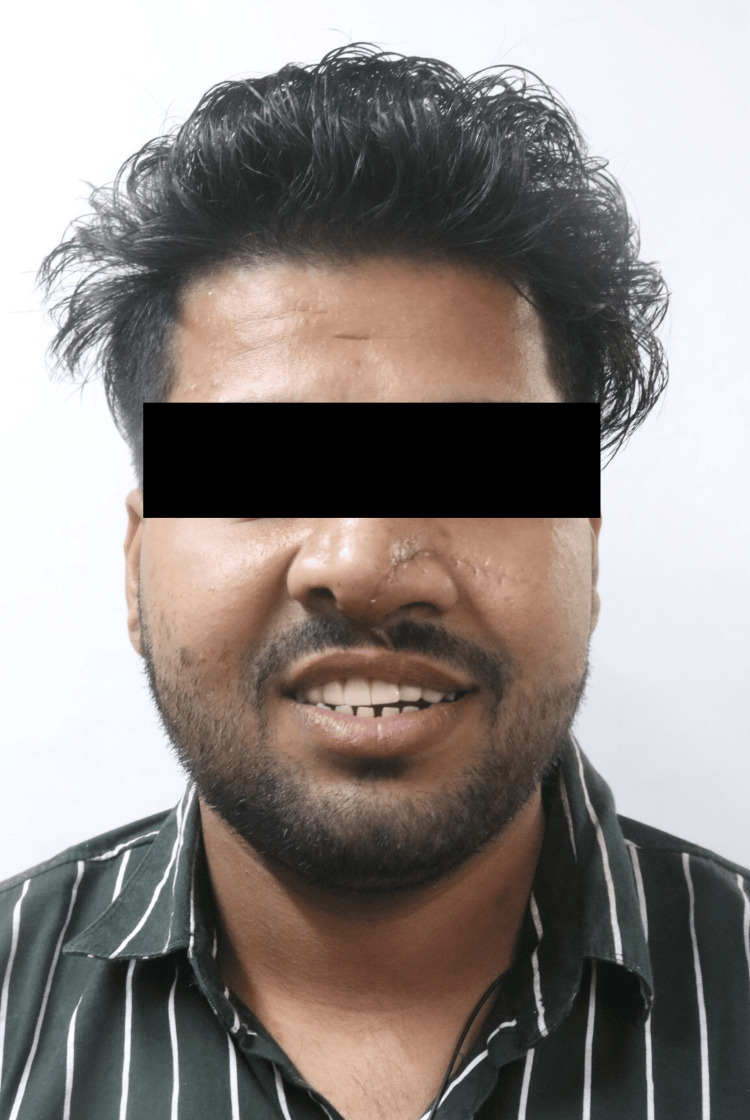
Extraoral frontal view with Andrew's bridge Extraoral frontal view along with inserted Andrew's bridge system.

**Figure 18:**
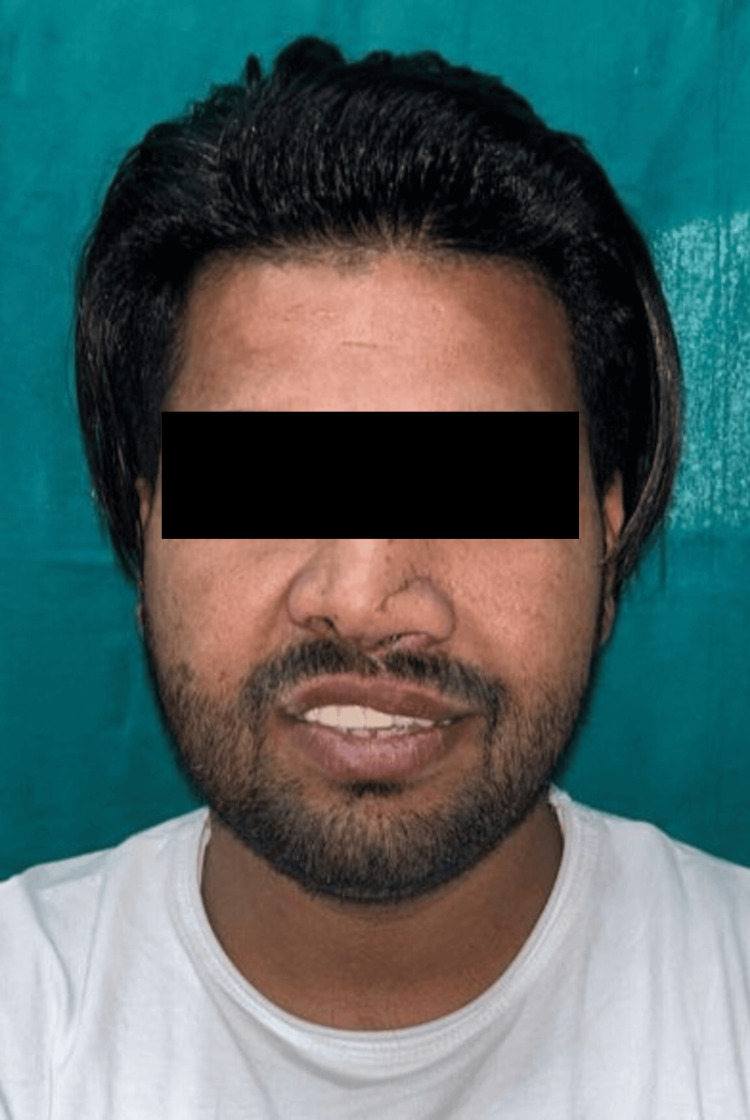
Follow-up after one year Extraoral frontal view on the follow-up visit of the patient for patient satisfaction and hygiene checkup.

**Figure 19:**
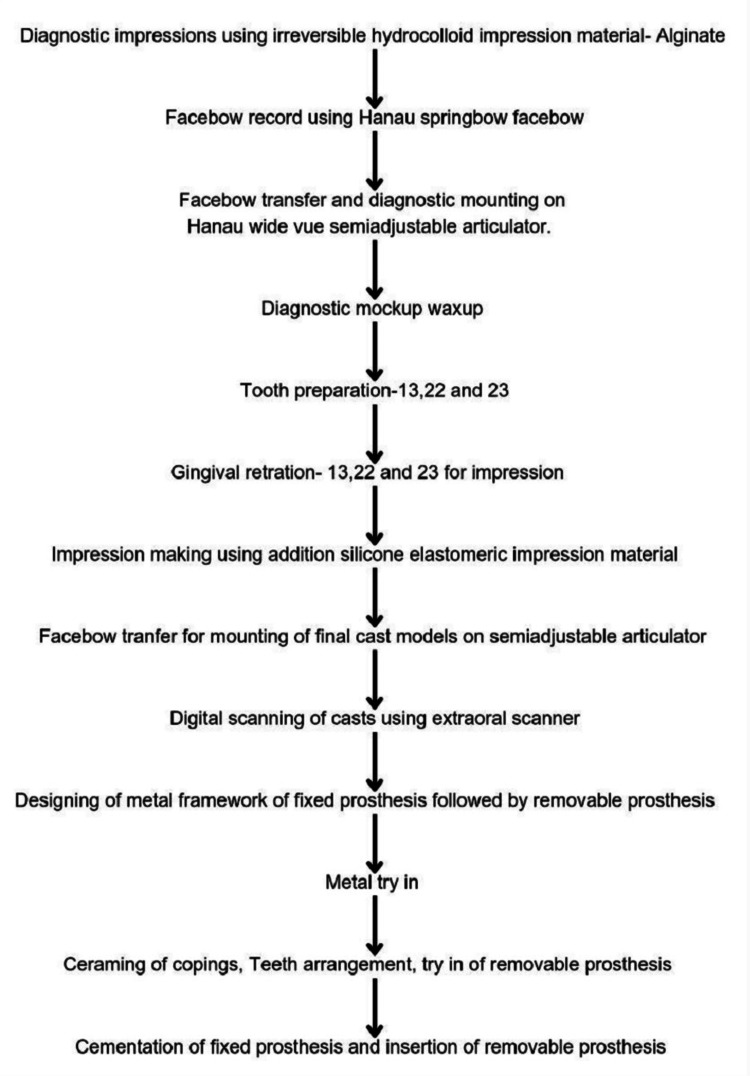
Flowchart of steps in the fabrication of Andrew's bridge prosthesis. Steps in the dental technique for the fabrication of Andrew's bridge system.

The cast partial denture was milled using a Co-Cr alloy disc. The smile line as well as the high and low lip lines were measured using the smile creator tool in Exocad software. Teeth arrangement on the cast partial denture was followed by a try-in to check for anterior guidance, then acrylic resin polymerization and gingival masking were performed. Upon completing the fabrication of both the fixed and removable portions of the prosthesis, the fixed prosthesis was cemented, followed by the insertion of the cast partial denture (Figure 16). The patient was satisfied with the final outcome, achieved in very few visits, with pleasing aesthetics and phonetics (Figure 17). The patient was recalled after three days, three months, six months, and one year (Figure 18), followed by yearly follow-ups to check for hygiene and compliance with the prosthesis. A flowchart (Figure 19) briefly explains the steps in fabricating the modified Andrew’s bridge system.

## Discussion

It has been documented that the majority of cases involving anterior tooth loss also exhibit alveolar bone loss, known as ridge defects, with 91% of patients showing such defects. Siebert (1983) introduced a classification system for ridge defects to evaluate deficiencies in form, function, and aesthetics. This system includes three categories based on the extent of the bony defect [5]:

Class 1: Buccolingual loss of tissue.

Class 2: Apicocoronal loss of tissue.

Class 3: Combination of buccolingual and apicocoronal loss of tissue.

The most frequently observed defects are combined Class III defects (seen in 56% of cases), followed by horizontal defects Class I (33% of cases), with vertical defects found in 3% of patients [6]. Depending on the severity of the defect, various treatment options can be considered, such as surgical bone augmentation techniques like cortico-cancellous onlay block bone grafting, distraction osteogenesis, guided bone regeneration, or ridge expansion, followed by prosthetic rehabilitation [7]. For substantial anterior ridge defects that cannot be solely addressed with surgical augmentation, Andrew's fixed-removable system presents a commendable alternative. This approach was also endorsed by Mueninghoff et al. and Everhart et al. in their respective case reports [8, 9].

The Andrew’s bridge offers several benefits such as it minimizes the bulk of the denture, provides excellent retention, and allows for the replacement of missing alveolar structures. It ensures patient comfort, is cost-effective, and eliminates the need for palatal or lingual flange coverage typical of conventional removable prostheses. Additionally, it prevents impingement on soft tissues and surrounding structures, and acts as a stress breaker by distributing unwanted leverage forces [10].

The Andrew’s bridge also has some drawbacks: the procedures involved are technique-sensitive, and food accumulation in the flange area can lead to tissue proliferation where the bar contacts the ridge. Inadequate soldering can result in prosthesis failure. Additionally, it is not suitable for cases where the abutment teeth are periodontally compromised. Proper functioning requires a minimum occluso-gingival height of 3-4 mm.

The Andrew's bridge offers enhanced stability and retention due to its complete reliance on teeth for support, ensuring that occlusal forces align with the long axis of supporting teeth. The contouring of the pontic assembly's flange not only enhances comfort, aesthetics, and speech but also provides resistance against potential torque during functional activities. Moreover, a significant advantage of the Andrew's system is its ability to remove the pontic assembly for ease of hygiene procedures and to adjust it as the ridge undergoes resorption.

Modifications in Andrew’s bridge

Taylor and Satterthwaite introduced an alteration to the traditional Andrews fixed dental prosthesis aimed at replacing both soft and hard tissues following the resection of an odontogenic myxoma in the posterior mandible [11]. Their modification involved extending the bar gingivally to improve retention for the removable prosthesis. In a separate study, Rathee et al. proposed a modification to enhance prosthesis retention by replacing the prefabricated bar and sleeve attachment of the conventional Andrew’s system with two small disc-shaped magnets [12]. Additionally, Kumar et al. devised a modification to the Andrew’s bridge by extending the removable component into adjacent quadrant, thereby augmenting the cross-stabilization of the prosthesis [13].

In this case report, a modification was made to Andrew’s Bridge by incorporating an attachment on the palatal aspect of the bar. This addition prevents antero-posterior movement of the removable component, which can occur due to shear forces acting on the prosthesis in the buccolingual direction. Consequently, this modification enhances the stability of the prosthesis. In this adjusted design, the patrix is part of the fixed prosthesis, while the matrix is integrated into the removable prosthesis. Utilizing CAD-CAM technology reduced treatment time and improved the precision of the prosthesis. By attaching retainers to the bar, the common issue of inadequate soldering, which can lead to failure as reported in the literature, was eliminated. This approach allows for the fabrication of the entire fixed prosthesis as a single unit, thereby enhancing its structural integrity [14].

## Conclusions

It is essential to evaluate alveolar ridge deficiencies in edentulous individuals. These deficiencies can be classified according to Siebert’s classification, based on their severity. This classification helps in recommending various treatment options to ensure successful outcomes. In cases where traditional removable or fixed prostheses are not feasible, Andrew’s Bridge presents a viable alternative. This treatment option can effectively restore function, aesthetics, and speech while addressing the defect. Additionally, integrating digital techniques into the fabrication process can enhance efficiency and accuracy, saving time.
